# The Enigma of the Impervious Nobel Prize for Neurosurgeons

**DOI:** 10.7759/cureus.44246

**Published:** 2023-08-28

**Authors:** Oday Atallah, Mustafa Ismail, Aktham O Al-Khafaji, Samer S Hoz

**Affiliations:** 1 Neurosurgery, Hannover Medical School, Hannover, DEU; 2 Neurosurgery, College of Medicine, University of Baghdad, Baghdad, IRQ; 3 Neurosurgery, University of Cincinnati, Cincinnati, USA

**Keywords:** neurology, neurosurgery, scientific achievement, neurosurgeons, nobel prize

## Abstract

The Nobel Prize, which has been highly esteemed since its establishment in 1901, carries significant status within the scientific community. The Nobel Prize in Physics, Chemistry, Medicine, and Economics has long been recognized for its recognition of significant scientific contributions. However, the Nobel Prize in Physiology or Medicine holds a distinct significance due to its direct association with advancements in human health. The subject of neurosurgery, which encompasses both clinical and academic domains, has witnessed remarkable developments; nonetheless, it has not yet been awarded a Nobel Prize. The objective of this investigation is to elucidate the factors contributing to the enigmatic nature of this recognition and propose feasible techniques that can be implemented to achieve it.

## Editorial

Since it was first awarded in 1901, the Nobel Prize has earned a reputation as one of the most prestigious and well-known scientific honors in the world [1]. For decades, its winners have captivated the academic, creative, and political elites around the globe.

The Nobel Prizes in Physics, Chemistry, Physiology or Medicine, and Economics have all done exceptionally well as a means of recognizing the pinnacle of scientific achievement. The Nobel Prize in Physiology or Medicine, due to its close relationship to human health and disease, has attracted significantly more public attention than its peers in other disciplines, ensuring the lasting fame of its laureates. The science prizes were originally given out to single individuals; today, however, a maximum of three laureates per year can receive the award, for a total of just 12 per year [2].

Historically, neurosurgeons have been defined by their excellence in both clinical and academic work. Despite the high academic yield of the neurosurgical community, the Nobel Prize has long eluded its members. Even though they were nominated for the laureate, neurosurgery pioneers like Victor Horsley, Walter Dandy, and Harvey Cushing, to name a few, who made revolutionary contributions that went far beyond their relatively narrow field of practice, did not end up being named the winner [3]. Furthermore, many neurosurgical subspecialties have made great strides. Disciplines like cerebrovascular surgery, functional and stereotactic neurosurgery, and neuro-oncology have seen many advances in the past few decades, reflecting the exceptional dedication and research efforts carried out by their practitioners.

The extensive work and progress achieved by neurosurgeons since the establishment of neurosurgery as a specialty remain unrecognized by the Nobel Committee to this day. And so, a question begs to be asked. What are the reasons why the neurosurgeon was not awarded this prize, and what steps do we as neurosurgeons need to take in order to be recognized with a prize as prestigious as the Nobel?

Reasons pertaining to neurosurgery and neurosurgeons

Overlapping Expertise

Neurosurgery, the intricate art of exploring the human brain, frequently intersects with neurology, radiology, and oncology. The complex collaboration between these medical specialties makes it a daunting task for the discerning Nobel Committee to attribute singular achievements solely to the tenacious neurosurgeons. The remarkable progress in neurosurgery is frequently intertwined with progress in these related fields, making it difficult to distinguish between the specific contributions of each.

Clinical Versus Research Focus

Neurosurgeons serve as medical practice's sentinels due to their unyielding commitment to patient care and the creation of novel surgical procedures. The Nobel Prize, on the other hand, has always been viewed as a reward for the most innovative and forward-thinking scientific discoveries. There is an insurmountable gulf between neurosurgery's focus on patient care and the spirit of discovery that underpins the Nobel Prize. This can cause astounding advances in neurosurgical techniques to be overshadowed by other research-driven discoveries.

Complexity and Variability of Outcomes

Neurosurgery is a delicate tango with life and death, where every move leaves its mark on the patient and their complex neurological condition. Interventions in neurosurgery may have far-reaching and difficult-to-measure effects, in contrast to some scientific discoveries. As the Nobel Committee looks for a conclusive breakthrough deserving of the award, this inherent variability presents a challenge. Because of the wide variety and often individualized nature of neurosurgical procedures, it can be difficult to point to a single accomplishment that represents a truly groundbreaking contribution.

Longevity of Research Impact

As the Nobel Prize bestows on laureates an enduring legacy, the recognition of neurosurgical achievements requires a historical perspective. Neurosurgical breakthroughs often have far-reaching effects, but it may be years or even decades before we see the full extent of those effects. The Nobel Committee must navigate the complexities of identifying research that not only pushes the boundaries of knowledge but also has a lasting impact on medical practice.

The Youthful Field of Neurosurgery

In comparison to its medical counterparts, neurosurgery remains a young and vibrant field. While forefathers such as Victor Horsley, Walter Dandy, and Harvey Cushing laid the groundwork, it takes time for a discipline to mature enough to be considered for the Nobel Prize. The relatively recent establishment of neurosurgery as a formal medical specialty may have contributed to the limited number of neurosurgeons among past Nobel laureates.

Ethical Considerations

Neurosurgeons often face moral quandaries when they engage in controversial procedures like psychosurgery or perform experimental procedures. The Nobel Committee needs to be cautious as it evaluates candidates in order to avoid making any ethical missteps. A neurosurgeon's chances of winning the Nobel Prize may be affected by how society views the ethical dilemmas they've faced in their work.

Reasons relating to the scientists' participation in the ultimate decision

The Human Element of Decision-Making

The Nobel Prize is just a window into the complex network of human thought that lies behind it. Experts from the scientific community, the medical community, and the decision-making community all play a role in evaluating candidates. These subjective factors will undoubtedly influence the course of the Nobel Prize. The Nobel Committee's choices are not made in a vacuum but rather reflect the members' unique perspectives, biases, and experiences.

Perception of Neurosurgery as a Specialty

The recognition hinges on how the Nobel Committee understands neurosurgeons' work and defines neurosurgery as a field of medicine. If this mystery can be solved, neurosurgery's embrace of the Nobel Prize may finally be revealed. The perception of neurosurgery as primarily a clinical specialty may influence its recognition in research-driven awards like the Nobel Prize in Physiology or Medicine.

Impact of Geographic Bias

Decisions about who should win the Nobel Prize can be influenced by international politics. The prevalence of other medical specialties or the emphasis placed on particular geographical areas may have an impact on the recognition of neurosurgery. The distribution of Nobel Prizes across various regions and medical disciplines may reflect underlying geopolitical dynamics.

Lack of Broad Public Appeal

Although neurosurgical advancements have a significant impact on patient's lives, they may not always capture the public's imagination in the same way as other scientific advances. Public enthusiasm and support for a Nobel laureate's work can have an impact on whether or not that person receives the award. In order to convince the general public of the importance of their work, neurosurgeons may have to overcome some obstacles.

Historical Precedence

Inadvertent biases can be introduced by looking at the past distribution of Nobel Prizes in physiology or medicine. It's possible that the widespread lack of neurosurgeons among Nobel laureates has contributed to the misconception that neurosurgery isn't at the forefront of medical innovation. This historical precedent can influence the committee's perceptions and preferences.

What has to be done to get the Nobel Prize

An Institutionally Driven Strive for Excellence

The science Nobel Prizes serve not only to honor individuals but have also been an unspoken metric to evaluate the quality of universities and research institutions. Scientific establishments that have nurtured Nobel laureates and their breakthroughs are credited and held in high esteem [2]. As such, neurosurgical institutions should seek to invest in the academic and research endeavors of their members, as the return for that investment could be of extreme value to their international renown.

Establishing More Grand International Awards Within the Neurosurgical Community

Investigations focusing on the Nobel Prize in Physiology or Medicine in the past 30 years have shown a trend in the selection of laureates. Individuals who held the aforementioned prize had already established a portfolio of prestigious awards. Such track records aid the prospective laureates in gaining attention from the concerned international communities. International Neurosurgical bodies of authority should therefore put greater efforts into providing the neurosurgical community with a roster of highly prestigious awards within the field.

Changing the Classical Concept About the Origin of the Spark Idea, Its Innovation, and How to Explore Its Social Relevance

Besides a track record in laboratory research, scientists in positions of authority and neurosurgeons, in particular, must develop new moral virtues besides the traditional ones connected with bench work, such as meticulousness, trustworthiness, and selflessness. Typical lab sites are not the only places where creativity becomes decisive and grand ideas flash up. This may also happen at airports and international conferences, or during board meetings and committee gatherings.

Maintaining Free Movement of Highly Qualified Scientists in the Field of Neurosurgery Between International Institutions

This is to promote their work and improve the maturation of their projects. A prominent trend in the field of neurosurgery has been the tendency of neurosurgeons to settle in a single institution, cementing their presence in its foundations and building their own “kingdom.” Mobility analysis made clear that most of the Nobel laureates were mobile either after obtaining their academic degrees or after conducting their prize-winning work. In most cases, researchers move from one institution to another within the same country [4].

A Prize-Winning Mentality for the New Generation of Mentors

Remarkable connectedness among Nobel laureates is found through generations of mentoring relationships in the Academic Tree network, supporting the notion that assortative processes are at work in mentor-mentee selection. At least one Nobel laureate, Krebs (1967), attributes much of his success in science to his academic mentor [5]. The young and upcoming generation of mentors within the field of neurosurgery needs to cultivate academic thought in their mentees, driving and encouraging them towards developing the attitudes and skills needed for their work to be recognized by the scientific community.

Better Control of Neurosurgical Research Citation

The typical project to win the Nobel prize is usually a heavily cited one. Yet, the large volume of academic work published yearly in the field of neurosurgery negatively impacts the chance of great academic work being recognized. Intervention at this point is necessary, lest these exceptional efforts be drowned in a sea of publications.

Selfless, Supporting Attitudes of the Scientific Community

Nobel laureates have more Nobel laureate ancestors, more local and global academic descendants, and more local academic family members than do those who have not won the award [5]. With the recent rise of globalization brought about by the internet and the unlimited scientific resources and means of communication it provides, the establishment of international academic networks in neurosurgery has become a necessity of the modern era. Not only would this help disseminate and flourish innovative ideas in science [5], but also allow for far-distance mentorship and guidance.

National Support for Neurosurgical Initiatives

Obtaining Nobel Prizes constitutes a crucial challenge for nations worldwide, as they are a significant determinant of a country’s prestige and a reliable index of the efficiency of its scientific policy [1]. As the field of academic neurosurgery continues expanding as a sandbox for creativity and innovation, investing in its progress at a national level could prove lucrative in the long term.

In conclusion, the elusive Nobel Prize in neurosurgery remains an audacious quest that demands collective efforts and unwavering dedication from the neurosurgical community. Thus, more exploration of new initiatives is needed; both for individual neurosurgeons and for the whole neurosurgical scientific community as a whole. A final goal of our community would be promoting the development of performance-indicating algorithms for the future neurosurgical Nobel laureate.
